# Prevalence and risk factors associated with adverse birth outcome in Ethiopia: systematic review and meta-analysis

**DOI:** 10.4314/ahs.v25i4.13

**Published:** 2025-12

**Authors:** Teka Girma, Dessalegn Wirtu, Gudina Egata, Jote Markos

**Affiliations:** 1 Department of Public health college of health sciences and referral hospital, Ambo University, Ethiopia; 2 Department of Public health Institute of public health, Wollega University, Ethiopia; 3 Department of Nutrition and Dietetics, school of public health, college of health sciences, Addis Ababa University, Addis Ababa city, Ethiopia

**Keywords:** Prevalence and risk factors associated with adverse birth outcome in Ethiopia: systematic review and meta-analysis

## Abstract

**Introduction:**

Adverse pregnancy outcomes represent a major public health challenge in developed and resource-limited countries. Globally, approximately 60–80% of neonatal deaths occur in low birth weight infants, and more than 2 million infants die before birth each year and the burden of the problem is significant in Ethiopia. Therefore, the aim of this study was to determine the combined incidence and risk factors for poor birth outcomes in Ethiopia.

**Method:**

International databases (PubMed, Google scholar, web of science and scopus) were searched. A funnel plot and Begg test were used to see the publication bias. The heterogeneity of studies was checked using I-square statistics with a cut of point 75% and the Newcastle Ottawa (NCO) quality assessment tool was applied to ensure the quality of the included articles. A random-effect model was applied to pool the adverse birth outcome. The sub-group analysis and Meta-regression analysis were conducted by region in the country and year of publication to control heterogeneity and to show variation.

**Result:**

A total of 16636 study participants were used to estimate the pooled prevalence of adverse fetal outcomes. The overall pooled prevalence of adverse fetal outcomes in Ethiopia was 28 (95% CI; 24-32; 12 = 97.44 percent, Pv= 0.001). Low birth weight 10.06% (95% CI; 7.21–12.91) and preterm birth 8.76% (95% CI; 5.4–12.11) were the most common adverse birth outcome at the national level. Rural in residency (AOR = 2.31; 95% CI: 1.64–3.24), lack of antenatal care follow up (AOR = 3.84; 95% CI: 2.76–5.35), pregnancy-induced hypertension (AOR = 7.27; 95% CI: 3.95–13.39), advanced maternal age ≥ 35(AOR = 2.72; 95% CI: 1.62–4.58, and having current complication of pregnancy (AOR = 4.98; 95% CI: 2.24–11.07) were the factors associated with adverse birth outcome.

**Conclusion:**

The pooled prevalence of adverse fetal outcomes in Ethiopia was high. Rural in residency, lack of antenatal care follow up, pregnancy-induced hypertension, advanced maternal age ≥ 35, and having current complications of pregnancy were the factors associated with adverse fetal outcome

**Systematic review registration:**

Identifier: CRD42022327072

## Introduction

Adverse birth outcome which attributes to most perinatal deaths is an important indicator of child health and survival. Millions of newborns worldwide are affected by unfavourable birth outcomes. Each year, preterm delivery causes one million newborn deaths^1^,^2^. Every year, an estimated 15 million babies are born before 37 weeks of pregnancy. In Africa and South Asia, premature births account for more than 60% of all births^3^. Low birth weight (LBW), which is estimated to account for 15% to 20% of all births globally^4^, continues to be a critical public health issue, with the majority of these deaths taking place in sub-Saharan Africa^1^,^5^,^6^. Pregnancy and its related problems contribute to a significant proportion of reproductive mortality with maternal mortality is unacceptably high.

In Ethiopia, the burden of poor birth outcomes is significant^7^. Every year, about 320,000 infants are delivered before 37 weeks of pregnancy^1^. In 2014, there were 27,243 fatalities attributable to LBW, making up 4.53 percent of all fatalities^8^. Recent researches from various regions of the nation indicate that between 18.2% and 32.5 percent of births in Ethiopia result in unfavourable outcomes. 15–18 LBW and stillbirth are the most typical types of poor delivery outcomes^9^,^10^. Induced labor, hypertensive disorders of pregnancy, antepartum hemorrhage, premature rupture of membranes, prior poor obstetric history, multiple pregnancies, multigravida, insufficient antenatal care (ANC), hemoglobin level 11 g/dL, rural residence, and malaria infection during pregnancy are all significantly linked to poor birth outcomes, according to a number of studies^9^-^13^.

Premature birth and low birth weight (LBW) are consequential factors that kick in to perinatal and neontal mortality and have negative longterm health backwash for survivors, especially in low- and middle-income countries (LMICs)^14^,^15^. Although adverse birth outcomes and their consequences are preventable^14^,^16^,^17^, they are particularly common in low- and middle-income countries. This means that a significant proportion of infant mortality is due to premature births. 15.4% of the 6 estimated. The three million children less than 59 months who died in 2013 were due to complications related to premature birth. The most common cause of death in newborns (death within the first 28 days of life) is premature birth^14^. Likewise, the risk of postnatal and perinatal mortality (stillbirths and newborns) is higher in children with low birth weight^15^.

Having the burden of adverse birth outcomes and key risk factors will provide policy makers and healthcare practitioners working in the region with evidence that can be used to inform strategies to achieve reductions in adverse birth outcomes and improve overall perinatal health. These research findings will help to design targeted interventions and better allocate resources to where they are needed. Additionally, findings of the review will inform future etiological research on the effect of risk factors of adverse birth outcomes in the region

## Methods

**Figure 1:**
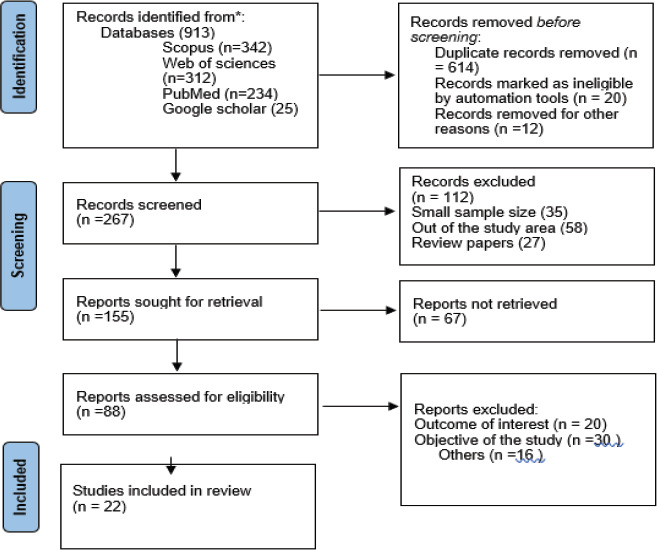
PRISMA flow diagram on adverse pregnancy outcome in Ethiopia, 2022^14^

This systematic review and meta-analysis was conducted to estimate the pooled prevalence of adverse fetal outcomes, the most common magnitude of adverse fetal outcomes and associated factors in Ethiopia using the standard PRISMA checklist guideline (Fig 1.)

### Reporting

This systematic review and meta-analysis was designated to estimate the pooled prevalence of adverse birth outcomes and associated factors among pregnant women in Ethiopia. The result is reported based on standard Preferred Reporting Items for Systematic review and Meta-analysis (PRISMA) checklist^15^. The review protocol was submitted for registration in the international prospective register of systematic reviews (PROSPERO) and registered with PROSPERO registration number (PROSPERO 2022: CRD42022327072)

### Database and search strategy

International databases which are Pub Med, Google Scholar, Web of Science, Scopus and different gray pieces of literature and articles published in the university online repository were included. Core searching terms were used using PICO formulating questions. These were: “newborn”, “adverse birth outcome”, “fetal outcome”, “stillbirth”, “low birth weight”, “neonate”, “prematurity”, and “congenital anomaly, congenital defect”, “preterm”, “preterm birth”. “Ethiopia”. The following Searching terms were applied: neonate OR newborn OR women OR infant OR child OR children AND “abnormal birth weight” OR “congenital defect” OR “congenital anomaly” OR “stillbirth” OR “prematurity” OR “preterm birth” OR “low birth weight” OR “perinatal” OR “neonatal death” OR “preterm” AND Ethiopia and related terms. The search strategy has been employed from June 3/2022-June30/2022.

### Research question

What is the pooled prevalence of adverse pregnancy out come in Ethiopia? Which adverse birth outcomes are common in Ethiopia? What are the factors affecting the adverse birth outcome in the area?

### Study selection

Initially, all articles were exported into Endnote version 9 software and checked for duplication. The duplicated articles were removed. Two independent authors, TG and MB, have reviewed the title and abstract. Three authors; DW, GE, and TG have scanned the abstracts and full documents. The disagreement was handled based on established article selection criteria.

### Inclusion and exclusion criteria

Twenty two Observational studies (case-control, cross-sectional and follow up study) which were previously published were included in this systematic review and meta-analysis. Articles reported the prevalence or/and a minimum of one contributing factor for adverse fetal outcomes is included. Only English language literature and research articles were included. Whereas, articles without full abstracts or texts and articles reported out of the outcome interest were excluded.

### Quality assessment

Two authors (TG & GE) independently assessed the quality of each study using the Newcastle-Ottawa quality assessment (NOQA) form was used. Any disagreement was resolved by the hindrance of the third reviewer (MB). The following NOQA items used to appraise case-control studies were: [1] comparable groups, [2] appropriateness of cases and controls, [3] criteria to identify cases and controls, [4] standard measurement of exposure, [5] similarity in the measurement of exposure for cases and controls, [6] handling of confounder [7], strategies to handle confounder, [8] standard assessment of outcome, [9] appropriateness of duration for exposure, and [10] appropriateness of statistical analysis. Items used to appraise cross-sectional studies are: [1] inclusion criteria, [2] description of study subject and setting, [3] valid and reliable measurement of exposure, [4] objective and standard criteria used, [5] identification of confounder, [6] strategies to handle confounder, [7] outcome measurement, and [8] appropriate statistical analysis. Therefore to consider the studies have low risk, the value should be 50% and above the quality assessment indicators

### Data extraction

After collecting findings from the entire database, the articles were transferred from Endnote version X9 software to the Microsoft Excel spreadsheet to remove duplicated studies. Two authors (TG and GE) independently extracted all the important data using a standardized Newcastle Ottawa data extraction format. Any disagreement between reviewers was resolved by the third reviewer (DW) through discussion and consensus. The name of the author, sample size, publication year, study area, region, the overall prevalence of ad outcome with its outcome categories with 95%CI and associated factors were collected. The reviewer contacted the corresponding author(s) for further information whenever pertinent data were missed from the included studies.

### Statistical analysis

Publication bias was checked using the funnel plot and Egger's regression test^16^. The heterogeneity of studies also computed using the Cochrane Q-test and I-squared statistic^17^,^18^. Pooled analysis was conducted using a weighted inverse variance random-effects model^19^. Subgroup analysis was also conducted using the study region and year of publication. STATA version 14 statistical software. Forest plot format was used to present the pooled point prevalence with 95%Cl. For associations, a log odds ratio was used to decide the association between associated factors and adverse birth outcomes (Figure 1).

## Result

### Characteristics of the included studies

A search approach that considered adverse pregnancy outcomes and related factors in Ethiopia yielded 913 publications in PubMed, Google Scholar, Scopus, an academic website, and other gray literature sources. After removing duplicates, two hundred sixty seven (267) studies remained. As a result, 155 full-text publications were retrieved and the inclusion criteria checked, leading to the removal of a further 88 articles, mainly for pragmatic reasons. Twenty-two studies eventually qualified for inclusion in the final systematic review and meta-analysis (Table 1)

**Table 1:** Study characteristics included in the systematic review and meta-analysis of adverse birth outcome in Ethiopia, 2022

3	Mekonen M. et al.	2018	Somali	Fafan	545	crossectiona	1050	51.9	preterm birth, congenital defect
**4**	Tsegaye lolaso	2019	SNNPR	Kembata	100	crossectional	718	13.928	preterm, LBW, still birth
**5**	Yeshialem E, et al.	2019	Oromia	Jimma	86	case control	344		preterm, still birth, low birth weight
**6**	A Eshete et al.	2013	Amhara	North wollo	68	cross sectional	295	23.051	Preterm birth, LBW, Stillbirth & congenital defect
**7**	Ediris et al	2018	Oromia	Shashemene	50	cross sectional	306	34.967	PPH, hypertensive disorder
**8**	Abera Haftu et al.	2017	Tigray	Shire	96	cross sectional	425	22.588	Preterm birth, LBW, Stillbirth Low
**9**	Abebe Eyowas et al.	2017	Amhara	Bahirdar	1135	cross sectional	3003	37.8	Preterm birth, LBW, Stillbirth Low
**10**	Ritbano A et al.	2019	SNNPR	Butakira	57	Crosssectional	313	18.2	LBW, preterm, still birth, visible congenital anomalies
**11**	Daniel Belema F et al.	2019	Oromia	west shoa	171	case control	591		preterm, LBW, still birth
**12**	Feleke G meskel	2016	SNNPR	Gamogofa	158	case control	426		preterm, LBW, still birth, congental anomalies
**13**	Cherie N, et al.	2017	Amhara	Desse	150	crossectiona	462	32.5	preterm, LBW, still birth
**14**	Hailemichael et al	2020	Tigray	Tigray	135	case control	405		preterm, LBW, still birth
**15**	Degno et al	2021	Oromia	Bale	122	crossectiona	580	21	preterm, low birth weight, still birth, congenital anormalies
**16**	Bezawit Abeje Alem et al.	2022	Amhara	Bahirdar	123	crossectiona	371	33.2	LBW, preterm birth, stillbirth, and had visible birth defects
**17**	Tsegaye and Kassa	2018	Sidama	Awassa	106	crossectiona	580	18.3	perinatal death, preterm birth, macrosmia, low birth weight
**18**	Tadese M. et. al	2022	Amhara	Debrebrihan	941	crossectiona	2418	28.3	perinatal death, stii birth, malpresentation, lowbirth weight
**19**	Jida Ali Hassen et al.	2021	SNNPR		91	case control	365		preterm, Low birth weight
**20**	D. Addisu et al.	2021	Amhara	south Gonder	130	case control	441		preterm, low birth weight
**21**	Abadiga et al.	22	Oromia	west Ethiopia	165	case-control	495		Adverse birth outcome
**22**	M Tefera et al.	2021	Dirre, oromia, harari		471	follow up	2246	20.97	Adverse birth outcome

The included researches were from Oromia, Amhara, sidama, SNNPR (South Nation Nationalities people and representatives), Tigray, Somali, and Addis Ababa, among other regions of Ethiopia. Finally, this systematic review and meta-analysis included 22 papers, with a total of 16636 study participants. Among the included researches fifteen (15) were cross-sectional studies and 6 case-control studies and one study was follow up study design.

The author's name, year of publication, outcome of interest, total number of study participants, prevalence/proportion, and study area from the original studies were all included (Table 1). Amhara region was the one that report the high number of research papers, whereas the Somali and Sidama regions had the fewest that included in this systematic review and meta-analysis. Sidama is the newly emerged region in the country which was previously part of South Nation Nationalities people and representatives.

### Prevalence of Adverse pregnancy outcomes in Ethiopia

**Figure 2:**
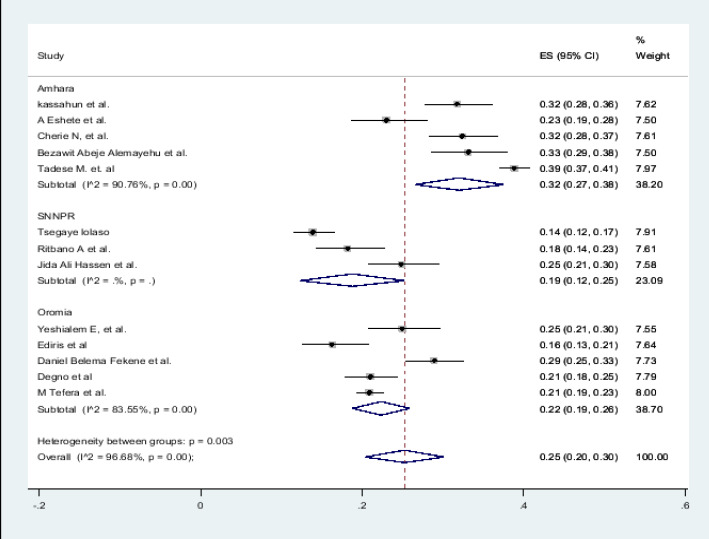
Forest pilot of the pooled prevalence of adverse birth outcome in Ethiopia, 2022

In the present systematic review and Meta-analysis drew on 22 studies, totaling 16636 study populations. A forest plot depicts the overall pooled prevalence of adverse fetal outcomes (Fig. 2). Despite the fact that the pooled estimated prevalence of adverse birth outcomes in Ethiopia was 25 percent (95 percent CI; 20-29; I2 = 96.00 percent, Pv= 0.001), the magnitude of each adverse neonatal outcome is as follows: low birth weight (9 percent), preterm birth (8 percent) and stillbirth (5 percent).

### Pooled meta-analysis of different adverse fetal outcomes categories in Ethiopia

#### Pooled prevalence of low birth weight

**Figure 3:**
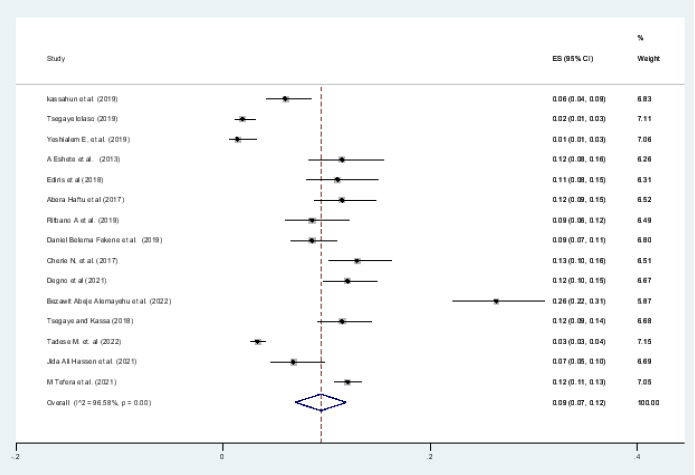
Forest pilot of the pooled prevalence of adverse birth outcome (Low birth weight) in Ethiopia, 2022

A forest plot depicts the quantified prevalence of low birth weight (Fig. 3). The overall pooled prevalence of low birth weight was 9 percent (95 percent CI: 7.0-12.0; I2= 96.58 percent, pv= 0.001). The included studies in this systematic review and meta-analysis were markedly heterogeneous since I2 was 96.66%

#### Pooled prevalence of preterm birth

**Figure 4:**
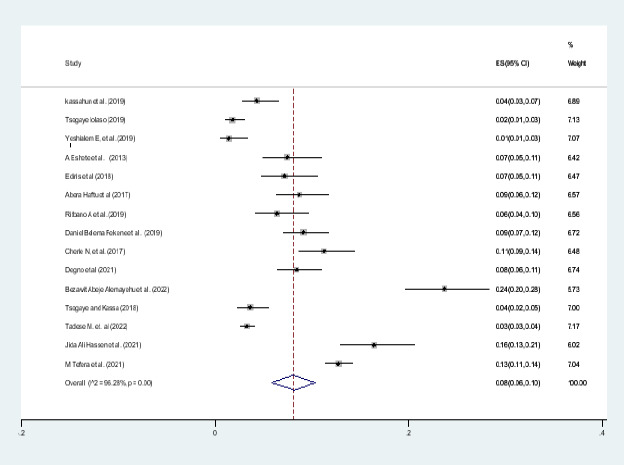
Forest pilot of the pooled prevalence of adverse birth outcome (preterm) in Ethiopia, 2022

A forest plot depicts the quantified prevalence of preterm birth (Fig. 4). The overall pooled prevalence of preterm was 8 percent (95 percent confidence interval: 6.0-10; I2= 96. percent, pv= 0.001). The included studies in this systematic review and meta-analysis were markedly heterogeneous.

#### Pooled prevalence of still birth in Ethiopia

**Figure 5:**
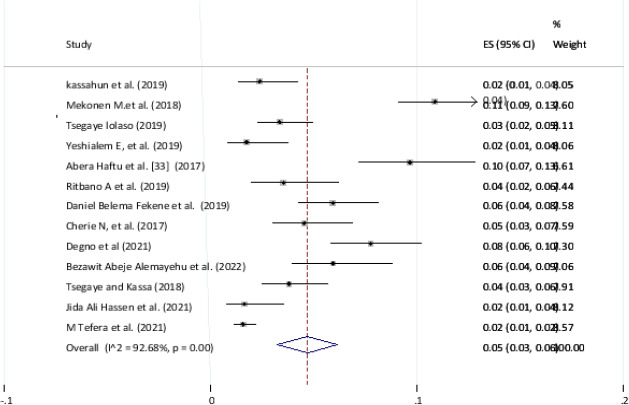
Forest pilot of the pooled prevalence of adverse birth outcome (still birth) in Ethiopia, 2022

The estimated prevalence of fetal death is presented in a forest plot (Fig. 5). The overall pooled prevalence of fetal death was 5 (95% CI; 3–6; I2 = 92.68%, p < 0.001). In this systematic review and meta-analysis, the included studies were characterized by marked heterogeneity (I2 = 92.68%, p < 0.001).

### Publication bias

**Figure 6:**
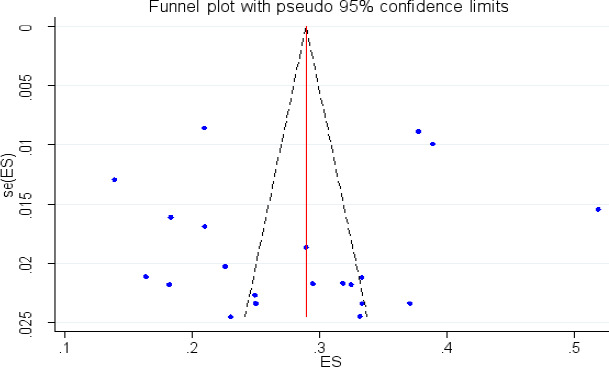
Funnel plot to show publication bias on adverse birth outcome in Ethiopia, 2022

A funnel plot was assessed for asymmetry distribution of adverse fetal outcomes by visual inspection and it was difficult to decide he presence of publication bias (Fig. 6). Unfortunately the Egger's regression test showed with a p-value of 0.35 showed that the presence of publication bias. Furthermore, low publication bias was detected using Egger's tests with a p-value of 0.03.

### Factors associated with adverse birth pregnancy outcome in Ethiopia

Multiple pregnancies, being anemic/Hgb level less than 11g/dl, having pregnancy complication during the current pregnancy, rural residency, lack of antenatal care follow-up, and advanced maternal age (greater than 35 years) were factors for adverse fetal outcomes in this systematic review and meta-analysis.

Women who had multiple pregnancies were about 5.8 times more likely to have an adverse birth outcome than mothers who had a single pregnancy (AOR 5.79 percent CI 1.11-10.48)

When compared to those who had a full ANC visit, pregnant women who did not have antenatal care follow-up to a health facility were 2.6 times more likely to have a negative fetal outcome (AOR=2.63; 95 percent CI: 1.44-3.81). Women who had current pregnancy complications (AOR=2.52; 95 percent CI:1.62-3.42) were nearly 2.5 times more likely to have an adverse fetal outcome than women who did not have current pregnancy complications.

Women in rural residency (AOR=2.09; 95 percent CI: 1.43-2.65), Women who lived in rural area were 2.3 times more likely to have a negative fetal outcome. The probability of developing adverse birth outcome for women whose their age was greater than 35 was about 2 times more when compared to their counter parts age less than 35.

### Subgroup analysis

**Figure 7:**
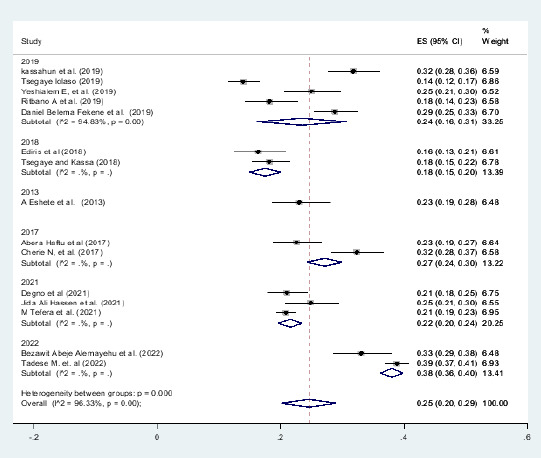
subgroup analysis by time on adverse birth outcome in Ethiopia, 2022

Subgroup analysis was employed with the evidence of heterogeneity. Therefore subgroup analysis was done by publication year and by region of Ethiopia and determinant factor. Hence the Cochrane I2 statistic =96.68%, P < 0.003) with evidence of marked heterogeneity between regions and Cochrane I2 statistic =96.33%, P < 0.001) with evidence of marked heterogeneity between years of publications (Fig 7).

## Discussion

Adverse birth outcomes such as low birth weight, stillbirth, and preterm birth constituted the highest rates of all the adverse pregnancy outcomes and are common in developing countries. In this systematic review and Meta-analysis, the pooled prevalence of adverse fetal outcomes in Ethiopia was 25 (95% CI; 20-39; I2 = 97.44, Pv= 0.001). The most common adverse birth outcome categories were low birth weight of 9 %^7^–^12^, and preterm birth 8%^3^–^6^ and still birth was 5%.

Birth weight is an important determinant of perinatal, neonatal and post neonatal outcomes. Poor intra-uterine growth would increase the risks of perinatal, infant and child mortality and morbidity. Consequently, addressing the burden of low birth weight would contribute significantly towards the achievement of sustainable development Goals. In the present study the magnitude of low birth weight was lower than study conducted on regional and global estimate by UNICEF^20^. This might be due to different socio cultural, socio economic and time difference. The current study mainly focused on one country and whereas the second studied on global.

This review was estimated the overall prevalence of preterm (prematurity) among adverse fetal outcomes in Ethiopia. Hence, the overall pooled prevalence of preterm was 8 %. This study finding was less than the study done in Asia 10.4, North America 11.2, Sub-Saharan Africa 12%, and Nigeria 11.4 but in line with study done South Korea 7.1% and Indonesia 10.4%^21^-^23^. This result is consistent with Asian and African countries as the health care system for maternal and newborn health are almost similar. In addition, Low income countries are implementing various prevention strategies and modalities for preterm births in collaboration with governmental and non-governmental organizations from African countries, including our country. This result made being lower compared to the global target for preterm births for the contribution of neonatal mortality and - morbidity.

This meta-analysis was estimated the national prevalence of stillbirth among adverse fetal outcomes in Ethiopia. Hence, the overall pooled prevalence of stillbirth was 7.09% (4.93–9.26). This review finding is lower than the study conducted in India which was 25.3, Pakistan which was 56.9% and Guatemala 19.9^5^,^24^. This discrepancy may be due to the fact that the study participants included in this systematic review and meta-analysis assessed multiple original studies in a single country; could be not global representative compared to studies that are conducted worldwide and consist of many countries at the same time.

Having pregnancy complications was more than two times more likely to develop adverse fetal outcomes. This study finding is in line with the study done in Kenya^25^, Bangladesh^26^, and China^27^. The possible reason could be due to women who have current pregnancy complications such as: Premature rupture of membranes, antepartum hemorrhage, and abnormal labor and pregnancy are the most common complications of pregnancy and childbirth, leading to preterm birth, stillbirth, and low birth weight.

The odds of living in rural were two times more likely to develop adverse fetal outcomes. This study finding is in line with the study done in China^27^, and urban Brazil^28^. This might be due to women who live in rural areas aren't getting health care services comprehensively and they are less likely to be informed about the danger sign and complication of pregnancy, labor, and delivery. Furthermore, cultural practices and norm in rural areas have a great effect on the nutritional status of women through the prevention of essential foods and or drinks^29^.

This review was estimated the overall prevalence of low birth weight among adverse birth outcomes in Ethiopia. Hence, the overall pooled prevalence of low birth weight was 10.06% (7.21–12.91). This study finding is in line with the study done in Indonesia 12.9], Armenia [9.0%, higher than the study conducted in Nigeria 6.3% and lower than the study done in Kenya 12.3], Tanzania (13.9%), South Africa 38.54%(30, 31). Low birth weight has different known and idiopathic risk factors; such as environmental and lifestyle risk factors, fetal risk factors, obstetric related factors, medical related factors, and maternal & family sociodemographic risk factors. Having the supremacof the above-motioned risk factors in each country may be increasing the magnitude of the preterm birth even death may have happened secondary to prematurity of the baby.

Having no ANC follow up was 2.6 times more likely to develop adverse birth outcomes. This study finding is in line with the study done in united republic of Tanzania^32^, and retrospective cross sectional study conducted in Gambia^33^. This might be due to During ANC follow up women will have a chance to access information related to danger signs of pregnancy. Having regular ANC follow up will also help a pregnant woman seek early treatment for her potential pregnancy-related problems.

Publication bias is one or more of the following has existed: selection bias, true heterogeneity, artifact, and chances are the main sources of publication bias. Large studies are likely to be published regardless of statistical significance because these studies involve large commitments of time and resources whereas Small studies are at greatest risk for being lost, because of the small sample size. In this study publication bias was not detected (Eggers, p value = 0.522) of the polled estimated prevalence of adverse fetal outcomes.

## Conclusion

The overall pooled estimate of adverse fetal outcomes in Ethiopia was high. Being resided in rural, not attending ANC, age ≥ 35, and developing complication during pregnancy and child birth were identified predictors for adverse fetal outcomes in Ethiopia. Therefore, based on the study findings, the authors recommend particular emphasis shall be given to have regular antenatal care follow up eight visits as per national recommendation, health education, early detection, and intervention of obstetric complications. Creating awareness of women on the effect of pregnancy at an advanced age, and providing timely and focused antenatal care (ANC) follow up to all pregnant women are very crucial to reduce the magnitude of the problem

## Data Availability

Data are available and the author is ready to provide when requested
